# Farthing Dinners

**Published:** 1887-03-05

**Authors:** 


### Farthing Dinners for Hungry Children.

By a Young Hand.

"We dread the coming winter," sigh the children in the street,
"For the cold it chills our bodies, and shoeless little feet.
About the shops we wander to the market down our way,
With eyes too tired for weeping, and hearts too sad to play.
We are hungry in the morning, and go starving to our bed,
And it can't be 'Jolly Christmas' when we want a bit of bread;
We may cry for food to mother, she'll have nothing left to give
In the long and dreary winter that is coming—if we live!"
*Punch*, 27th Nov., 1886.

Through "the long and dreary winter" that is now, let us hope, well-nigh spent, Mr. G. H. Sargant has worked with brave and patient kindness to alleviate the woes of the hapless little ones of the great smoky capital of the Midlands. In the hope of showing the way to other would-be helpers of the starving children in our large towns, Mr. Sargant has published a pamphlet, entitled *Farthing Dinners*, giving an account of the management and arrangements of his Birmingham soup-kitchens, and recipes for the best-liked and most economical combinations. Most of these recipes include leguminous and farinaceous materials only, which are found more digestible and more nutritious for the badly fed class of children who benefit by them than any meat diet could be. Indeed, Mr. Sargant says, "Abundant, rich, and highly-concentrated food, though nutritious to the well-fed, is almost poison to those long restricted to few, poor, and irregular meals; it throws their digestive organs completely out of gear, and when not absolutely harmful it is merely hurried through the system without benefit. To succeed in any work amongst a class whose thoughts, words, and works offer not one point of resemblance to our own, we must begin by giving them what they themselves want, not what we think they ought to want. Having learnt this—and it is no easy lesson—we must next learn what they do want. The first and most astonishing fact one learns, when trying to feed the poor, is, that they, or at any rate their children, cannot and will not eat meat; and that the longer they have been on the verge of starvation the less meat they can eat. It may be taken, I think, almost as an axiom in this kind of work, that no children who can pay a halfpenny a day regularly out of their own resources, and can take strong food without injury, are deserving objects for charity." The suggestion offered in this last sentence, coming from a gentleman of such wide experience as Mr. Sargant, is well worthy of note by all interested in helping the really indigent, and discouraging those (a numerous class, we are afraid) who will take advantage of any and every form of charity, whether they stand in need of assistance or not. "When a large portion of the children cannot pay," Mr. Sargant proceeds, "a system must be introduced of distributing free tickets or orders. In this case, the difficulties will be, when funds have been provided, to reach the really necessitous cases, and to prevent all the tickets being given away at once. If a week's supply is distributed to the children, the kitchen will be swamped for a day or two and then empty; the children seem quite unable to save anything for more than twenty-four hours. On one point I would lay very particular stress: money or tickets must never be given to unknown children, especially at or near the kitchen. After a case or two of promiscuous charity, I have seen many a child stand half-an-hour shivering in the cold in hope of a free ticket, and at last, finding it useless, produce his money, which has been in his pocket all the time. It is very hard for us to grasp and realise the value which the very poor attach to the smallest of coins." Mr. Sargant considers that the school teachers to whom the children are personally known, and the visiting-officers who are acquainted with the circumstances of the parents, are the best mediums for distribution. It has been found, by long and careful experiment, that good nourishing and appetising soup, having for its basis lentil or haricot beans and split-peas, can be made at a cost of less than three halfpence a gallon, and so half-a-pint can be supplied to the children, together with a round of bread weighing one-tenth of a pound, spread with a quarter of an ounce of jam (at 2½*d.* a lb.), for one farthing. The halfpenny and penny dinners contain potatoes and scraps of lean meat, but are not found one whit more popular than the cheapest kind. Here is one of Mr. Sargant's recipes:

*To Make Eleven Gallons of Soup at 1½d. a Gallon.*

| | |
|---|---|
| Lentils | 5 lb. |
| Indian Meal | 3 lb. |
| Scotch Barley (ground) | 3 lb. |
| Carrots and Onions | 3 lb. |
| Salt | 1 lb. |
| Pepper | ½ oz. |
| Dried Mint | |
| Water | 10 gals. |

The cooking is done in an apparatus which costs between £8 and £9, and is the invention of the Rev. W. Moore Ede. This consists of a cylindrical stewing-pan, which stands in a somewhat larger boiler of the same shape, the intermediate space being filled with water. The boiler rests on an iron flange, and the whole is fixed in a galvanised iron case packed with asbestos, to retain the heat, and fitted with a heavy lid. Heat is supplied from beneath by gas-burners, the fumes of which are carried off by a chimney. "The whole process is gradual, and the preparations singularly easy. The stewing-pan is placed in the boiler; water according to the recipe is poured into it, and it is fastened down; water is then poured between the boiler and the pan until the intervening space is full to within three or four inches of the top; the gas is then turned on full to boil the water, which will take about three hours, and in the meantime the solid ingredients are weighed out and put into the pan. . . . All this should be done in the afternoon, and when the water boils, one burner should be turned out, the other put as low as it can be with safety, and the water outside the stewing-pan replenished if much has boiled away. The apparatus is then left till the morning, when it is opened, stirred, seasoning added if necessary, and the whole brought to the boil again until an hour before dinner-time, when the gas should be turned out. The whole process takes from eighteen to twenty hours."

Mr. Sargant further gives a list of prices at which he buys stores, and a complete inventory of utensils needed. The total initial cost of the latter, including the cooker, he estimates at £25 to £30, for a kitchen at which four or five hundred are to be fed. Details as to the formation of committees and the orderly management of the kitchens, are fully and clearly given, and we strongly commend Mr. Sargant's pamphlet to the consideration of charitable organisations in other large towns. To feed the hungry, and so give life and strength to body and brain, without pauperising them, or destroying their self-reliance, is an object so desirable, that effectual means to its attainment must meet with approval from all.

